# Zoledronic acid-loaded HAp–SiO_2_–CaMoO_4_:Eu^3+^ with luminescent properties as a novel drug delivery system

**DOI:** 10.55730/1300-0527.3780

**Published:** 2025-12-26

**Authors:** Emine KUTLU, Muhammad Asim ALI, Fatih Mehmet EMEN, Canan VEJSELOVA SEZER, Hatice Mehtap KUTLU

**Affiliations:** 1 Department of Chemistry, Faculty of Arts and Sciences, Burdur Mehmet Akif Ersoy University, Burdur, Turkiye; 2 Department of Biology, Faculty of Arts and Sciences, Kütahya Dumlupınar University, Kütahya, Turkiye; 3 Department of Advanced Technologies, Eskişehir Technical University, Eskişehir, Turkiye

**Keywords:** Theranostic, drug delivery, hydroxyapatite, CaMoO_4_:Eu^3+^, zoledronic acid, Saos-2

## Abstract

CaMoO_4_:Eu^3+^-functionalized hydroxyapatite–silica (HAp–SiO_2_–CaMoO_4_:Eu^3+^) core–shell nanocomposites were synthesized for the first time and evaluated as a multifunctional drug delivery and imaging platform. HAp–SiO_2_ nanocomposites were prepared via a hydrothermal route and subsequently functionalized with a luminescent CaMoO_4_:Eu^3+^ shell using the Pechini sol–gel method. Structural analyses confirmed the successful coexistence of HAp, SiO_2_, and CaMoO_4_:Eu^3+^ phases, while electron microscopy revealed spherical core–shell morphologies. Dynamic light scattering measurements showed average hydrodynamic particle sizes of approximately 1085 nm for HAp–SiO_2_ and 1427 nm for HAp–SiO_2_–CaMoO_4_:Eu^3+^ nanocomposites, indicating particle clustering in aqueous media, which is consistent with the low surface charge of the particles. The Eu^3+^-doped shell exhibited a strong red emission centered at 615 nm, demonstrating suitability for luminescence-based imaging. Zoledronic acid (ZA) was efficiently loaded onto the nanocomposites under supercritical CO_2_ conditions, providing high loading efficiency and sustained release behavior. In vitro release studies in phosphate-buffered saline (pH 7.4, 37 °C) followed the Korsmeyer–Peppas kinetic model (n = 0.83), indicating a non-Fickian diffusion mechanism. Cytotoxicity assays on Saos-2 osteosarcoma cells demonstrated that ZA-loaded nanocomposites exhibited enhanced antiproliferative activity, with an IC_50_ value of 56.33 μM after 48 h. These results highlight the potential of HAp–SiO_2_–CaMoO_4_:Eu^3+^ nanocomposites as an integrated theranostic system for targeted bone cancer therapy. Overall, the results demonstrate that the proposed nanocomposite design successfully translates the intended theranostic concept into experimentally validated structural, optical, and biological performance.

## 1 Introduction

Osteosarcoma or osteogenic sarcoma is the most common primary malignant tumor of bone-forming cells. The peak incidence of osteosarcoma is seen in adolescence, although it can occur at any age and affect any bone, most commonly the knee and the proximal humerus [1]. For the treatment of osteosarcoma, different derivatives of bisphosphonates are applied. Bisphosphonates inhibit osteoclast-mediated bone resorption activity, potentially acting by preventing or delaying skeletal-related conditions in patients [2]. These medications work by reducing the osteoclast function to slow down bone turnover, improve bone density, and lower the risk of bone fractures [3]. Among bisphosphonates, zoledronic acid (ZA) is an intravenous third-generation amino-substituted bisphosphonate, which is a highly potent osteoclast inhibitor [4] and plays an important role in the treatment of bone metastasis, postmenopausal osteoporosis [3], and hypercalcemia of malignancy [5]. In addition, ZA has been shown to inhibit cellular proliferation and promote apoptosis in several tumor cell lines [4]. It has also been found to be effective in preclinical in vivo studies via the reduction of tumor size, growth, and metastasis. In combination therapy with ursolic acid [6] and oncolytic adenovirus [7], tumor development was inhibited by up to 50%. In addition, clinical studies revealed that substantial delay occurred in the progression of osteosarcoma [8]. Recent studies on the mechanisms of action of ZA indicated that inhibition of osteoclasts and cancer cells was achieved by the inhibition or suppression of differentiation receptors involved in the activation of nuclear factor κB ligand/receptor and the noncanonical Wnt/Ca^2+^/calmodulin dependent kinase II (CaMKII) pathway, blocking the differentiation of macrophages into osteoclasts. Moreover, apoptosis in osteoclast cells was induced by the inhibition of the farnesyl pyrophosphate synthesis-mediated mevalonate pathway and production of reactive oxygen species [9]. However, some side effects including headache, fatigue, hypocalcemia, bone and joint pain, and osteonecrosis have been observed with ZA. To avoid undesirable side effects and facilitate targeted therapy for osteosarcoma, various delivery systems such as hydrogels, nanocapsules, liposomes, bioceramics, and nanospheres have been utilized for the limited distribution of ZA [10]. Due to the enhanced permeation ability and higher retention effects of nanomaterials in drug delivery, these nanomaterials help achieve the accumulation of ZA in bone cells to disrupt the activity of mature osteoclasts by inhibition of cellular apoptosis [11]. Hybrid polymeric nanocomposites of poly(lactic-co-glycolic acid)-b-poly(ethylene glycol) and tocopheryl polyethylene glycol succinate loaded with high ZA payloads demonstrated promising cytotoxicity against the MCF-7 cell line [12]. It has been reported that ZA loaded by extrusion and precipitation method onto hydroxyapatite (HAp)-coated lipid nanoparticles provided sustained release behavior while inhibiting the progression of osteoporosis in the HFOb 1.19 cell line [11]. HAp has emerged as the most promising nanocarrier due to its high affinity toward bisphosphonates [13]. It is an inorganic, naturally occurring mineral form of calcium phosphate and the typical apatite lattice structure is (Ca_10_(PO_4_)_6_(OH)_2_). HAp has a composition and structure similar to those of human bone and teeth, and it plays an important role in physiological processes such as the regulation of calcium and phosphate levels in the body [14]. In addition, its biocompatibility and bioactivity make it an adaptable material for biomedical applications.

In this study, a novel Eu^3+^-doped CaMoO_4_ functionalized hydroxyapatite–silica (HAp–SiO_2_–CaMoO_4_:Eu^3+^) core–shell nanocomposite was successfully synthesized through a two-step process. The controlled synthesis of HAp–SiO_2_ nanocomposites often requires the use of surfactants and polymeric stabilizers. In this work, cetyltrimethylammonium bromide (CTAB) was used as a structure-directing agent to regulate the nucleation and growth of HAp on the silica surface, while polyethylene glycol (PEG) served as a stabilizer and cross-linking aid to limit aggregation during hydrothermal treatment. Such surfactant-assisted strategies enable the formation of well-dispersed and morphology-controlled HAp–SiO_5_ particles, which are essential for their subsequent functionalization with CaMoO_4_:Eu^3+^ and efficient zoledronic acid loading. Initially, HAp–SiO_2_ nanocomposites were prepared via the hydrothermal method. Subsequently, their surfaces were functionalized with a CaMoO_4_:Eu^3+^ precursor gel obtained by the Pechini sol–gel route. The resulting materials were calcined at 600 °C for 5 h to yield uniform core–shell nanostructures. Furthermore, ZA, a potent bisphosphonate, was efficiently loaded onto these nanocomposites under supercritical CO_2_ conditions, enabling controlled and sustained drug release.

To the best of our knowledge, this is the first report describing the synthesis of HAp–SiO_2_–CaMoO_4_:Eu^3+^ hybrid nanocomposites as dual-functional theranostic platforms. The integration of the luminescent CaMoO_4_:Eu^3+^ shell with the bioactive HAp–SiO_2_ core provides a synergistic system capable of simultaneous optical imaging and targeted drug delivery. The cytotoxic effects of ZA-loaded HAp–SiO_2_–CaMoO_4_:Eu^3+^ nanocomposites (HAp–SiO_2_–CaMoO_4_:Eu^3+^–ZA) were systematically evaluated on Saos-2 osteosarcoma cells using the 3-(4,5-dimethylthiazol-2-yl)-diphenyl tetrazolium bromide (MTT) assay in this study, confirming their antiproliferative potential and biocompatibility.

The main objective of this study was to develop a multifunctional core–shell nanocomposite that integrates (i) the bone affinity and drug-loading capacity of HAp, (ii) the structural stabilization provided by SiO_2_, and (iii) the luminescent imaging capability of CaMoO_4_:Eu^3+^ into a single theranostic platform. To achieve this goal, the structural, morphological, luminescent, drug-release, and biological properties of the synthesized HAp–SiO_2_–CaMoO_4_:Eu^3+^ nanocomposites were systematically investigated and directly correlated with their potential application in targeted osteosarcoma therapy. This innovative approach offers a new generation of multifunctional nanomaterials that combine imaging, therapeutic, and bioceramic features in a single biocompatible construct, thus providing a promising route for targeted bone cancer therapy.

## 2 Materials and methods

### 2.1. Materials

All chemicals purchased were of analytical grade and were used without further purification. Disodium hydrogen phosphate (Na_2_HPO_4_), calcium nitrate tetrahydrate (Ca(NO_3_)_2_·4H_2_O), polyethylene glycol 550 (PEG 550), calcium carbonate (CaCO_3_), europium oxide (Eu_2_O_3_), acetone, and nitric acid (HNO_3_) were obtained from Sigma-Aldrich (St. Louis, MO, USA). Hexadecyltrimethylammonium bromide (CTAB), tetraethyl orthosilicate (TEOS), acetic acid, toluene, sodium molybdate dihydrate, citric acid monohydrate, polyethylene glycol 300 (PEG 300), and ammonia were obtained from Merck (Darmstadt, Germany). Zoledronic acid (ZA) (4 mg/5 mL concentrated solution for intravenous infusion) was purchased from Polifarma (Tekirdağ, Türkiye).

### 2.2. Instrumental

The FT-IR spectra were recorded in the 4000–400 cm^−1^ range with an IRTracer-100 FT-IR spectrophotometer (Shimadzu, Kyoto, Japan). The phase analyses of the nanocomposites were carried out with an AXS D8 ADVANCE X-ray diffraction device (Bruker, Billerica, MA, USA). The particle size of the nanocomposites was determined with a Zeta-sizer Nano-ZS (Malvern Instruments, Malvern, UK). Surface morphologies of the particles were examined with JEOL JSM-7100-F SEM and JEOL JEM-1400 PLUS TEM devices (JEOL, Tokyo, Japan). A Cary Eclipse fluorescence spectrophotometer (Agilent, Santa Clara, CA, USA) was used for photoluminescence (PL) measurements. The PL spectra were obtained at room temperature. A PG Instruments UV-visible spectrometer (Wibtoft, UK) was used for absorbance measurements.

### 2.3. Methods

#### 2.3.1. Synthesis of HAp–SiO_2_ nanocomposites by hydrothermal method

Hap–SiO_2_ nanocomposites were synthesized by hydrothermal method in accordance with the literature [15]. CTAB (0.0063 mol) was dissolved in 37.5 mL of distilled water at room temperature and mixed with Na_2_HPO_4_ (0.009 mol) and incubated for 30 min. The mixture was adjusted to pH 4.5 with acetic acid. Ca(NO_3_)_2_·4H_2_O (0.0152 mol) was dissolved in 52.5 mL of distilled water and 5.45 mL of PEG 550 was added to the solution. This solution was stirred at room temperature for 20 min and Na_2_HPO_4_-CTAB was added dropwise to the first solution. TEOS (0.0096 mol) dissolved in acetic acid (0.2 mol) was then added to this mixture and it was stirred at room temperature for 30 min. This final mixture was adjusted to pH 11 with ammonia and placed in a Teflon-lined stainless-steel autoclave. The autoclave was heated at 200 °C for 24 h. The white suspension was taken out of the autoclave and centrifuged at 5000 rpm for 10 min. The resulting white precipitate was washed with ethanol several times and dried in a drying mill to obtain the final product.

In this step, CTAB was employed as a surfactant to direct the nucleation and growth of HAp on the SiO_2_ surface, ensuring the formation of rod-like and hexagonal nanostructures. PEG 550 was used as a stabilizer and cross-linking aid to minimize aggregation during the hydrothermal reaction (see Section 1 for more details).

#### 2.3.2. Preparation of CaMoO_4_:Eu^3+^ functionalized HAp–SiO_2_ nanocomposites (HAp–SiO_2_–CaMoO_4_:Eu^3+^)

The Pechini sol-gel method was used to prepare the CaMoO_4_:Eu^3+^ precursor. Eu^3+^ doping ions (5%) were used in accordance with the literature [16,17]. First, 3.0 mL of HNO_3_ was added to CaCO_3_ (0.19 g) and Eu_2_O_3_ (0.033 g) and mixed vigorously with a magnetic stirrer in the fume hood. To remove the HNO_3_ in the environment, the solution was heated and then 2.0 mL of deionized water was added. This process was repeated several times until the pH of the solution reached 2–3. In a separate beaker, 0.45 g of sodium molybdate dihydrate and 0.80 g of citric acid were added to 20 mL of a mixture of water and ethanol (15:5) and stirred until completely dissolved. This solution was added to the first solution and stirred for 20 min, and then 4.8 g of PEG was added to this solution as a cross-linker and mixed for 1 h to form a sol. HAp–SiO_2_ nanocomposites were added to the sol and mixed for 5 h. This suspension was centrifuged to separate the sol and the resulting precipitate was dried at 100 °C for 1 h. This solid product was calcined at 600 °C for 5 h to obtain HAp–SiO_2_–CaMoO_4_:Eu^3+^ nanocomposites with a core–shell structure. In this process, the well-dispersed HAp–SiO_2_ nanocomposites produced via surfactant-directed hydrothermal synthesis served as the core onto which the CaMoO_4_:Eu^3+^ shell was applied.

#### 2.3.3. Zoledronic acid loading onto HAp–SiO_2_–CaMoO4:Eu^3+^ nanocomposites by supercritical method (HAp–SiO_2_–CaMoO4:Eu^3+^–ZA)

Supercritical CO_2_ conditions were used to load ZA onto HAp–SiO_2_–CaMoO_4_:Eu^3+^ nanocomposites. First, 0.55 g of HAp–SiO_2_–CaMoO_4_:Eu^3+^ nanocomposites and 2.0 mL of ZA were loaded in a stainless-steel reactor. The reactor was kept at room temperature under 200 bar CO_2_ pressure for 2 h. The CO_2_ pressure in the reactor was then reduced with the help of the valve of the system. The resulting ZA-loaded nanocomposites (HAp–SiO_2_–CaMoO_4_:Eu^3+^–ZA) were collected from the bottom of the reactor with the help of a spatula [18–20].

### 2.4. In vitro ZA release studies

Drug release studies of ZA were performed by UV-Vis spectrophotometric analysis method. The calibration curve was prepared by reading the absorbance of ZA solutions prepared at different concentrations (1 mg/mL, 3 mg/mL, 5 mg/mL, 7 mg/mL, and 9 mg/mL) and calibration graphs are given in Supplementary Figure S1. The maximum absorbance value of ZA was determined as 210 nm. In vitro release studies were carried out in phosphate-buffered saline (PBS) solution at pH 7.4 and 37 °C, constituting the release environment. Next, 0.1 g of HAp–SiO_2_–CaMoO_4_:Eu^3+^–ZA was suspended in 25 mL of PBS solution. This suspension was stirred continuously with the help of a magnetic stirrer. At certain time points, 3.0 mL of sample was taken from the medium and centrifuged at 4500 rpm for 5 min, and the absorbance was read on the UV-Vis spectrometer. To maintain the total concentration, 3.0 mL of fresh PBS was added to the centrifuged portion and mixed, and the release medium was added again. The amount of ZA released into the environment was calculated with the help of absorbance values read at certain time points up to 36 h with a UV-Vis spectrometer at a wavelength of 210 nm. Drug release curves were created according to different kinetic models. The % cumulative drug release of the HAp–SiO_2_–CaMoO_4_:Eu^3+^–ZA nanocomposites was calculated. Drug loading efficiency was calculated according to Eq. (1) [16]:


(1) $$\begin{array}{l} \text{Loading efficiency }(\%)=[[(\text{Total amount of drug in nanocomposites})-(\text{Amount of drug on the surface of}\\ \text{nanocomposites})]/\text{Theoretical amount of drug in nanocomposites}]\times 100 \end{array}$$

### 2.5. Cell culture studies

#### 2.5.1. Cell culture

A human bone carcinoma (osteosarcoma) cell line was used in in vitro cytotoxicity studies of the HAp–SiO_2_–CaMoO_4_:Eu^3+^ and HAp–SiO_2_–CaMoO_4_:Eu^3+^–ZA nanocomposites. These commercially available cells were grown according to the manufacturer’s protocol. Cells were then placed in cryotubes and stored in liquid nitrogen at –196 °C. Cells to be used in experimental studies were thawed in a sterile water bath at 37 °C. Studies were carried out in a sterile laminar cabin. The cells were transferred to Falcon tubes containing Dulbecco’s modified Eagle medium (DMEM; comprising 20% fetal bovine serum, 1% penicillin/streptomycin, and 1% nonessential amino acids) using a pipette. The supernatant part of the cells was then centrifuged for 5 min at 1200 rpm and DMEM was added back to the medium. Cells were transferred to a T75-cm^2^ flask. Cells incubated in the incubator at 37 °C with 5% CO_2_ were passaged for a period of 3 days. After 6 passages, the cells were transferred to a 96-well plate. Cells incubated for 24 and 48 h were then checked with a tissue culture microscope.

#### 2.5.2. MTT method

The MTT method was used to evaluate cell proliferation. Cultured Saos-2 cells were planted in 96-well cell culture plates with 5 × 10^3^ cells in each well. The HAp–SiO_2_–CaMoO4:Eu^3+^ and HAp–SiO_2_–CaMoO4:Eu^3+^–ZA nanocomposites were diluted at concentrations of 31.25–1000 mg/mL. These concentrations were added to each well except the control well and the cells were incubated at 37 °C with 5% CO_2_ for 24 h. Subsequently, 20 μL of MTT dye (5 mg/mL) was added and the mixtures were incubated again at 37 °C and 5% CO_2_. The supernatant was removed and 200 μL of DMSO was added. The absorbance value was determined at ʎ_max_ = 570 nm with the help of a microplate reader. The group in which HAp–SiO_2_–CaMoO_4_:Eu^3+^ and HAp–SiO_2_–CaMoO_4_:Eu^3+^–ZA nanocomposites were not added was used as the control group. Using the obtained absorbance values, the IC_50_ concentration and the cell viability of the nanocomposites on the cells were calculated. Results were presented as dose-dependent % cell viability graphs given with error bars. In all graphs, *p < 0.5 indicates statistical significance. GraphPad Prism 6.0 (GraphPad Inc., La Jolla, CA, USA) was used for analysis, and one-way analysis of variance and the Tukey post hoc test were applied. Values of p < 0.05 were considered statistically significant. All experiments were done in triplicate and values of mean ± standard deviation were calculated. IC_50_ values were calculated and are provided below each graph.

## 3 Results and discussion

The primary objective of this study was to develop a multifunctional theranostic nanoplatform capable of simultaneous drug delivery and imaging for targeted osteosarcoma therapy. Accordingly, spectroscopic and structural analyses (FT-IR and XRD) are first discussed in this section to confirm the successful formation and phase integration of the HAp–SiO_2_–CaMoO_4_:Eu^3+^ core–shell architecture, which is essential for both drug loading and luminescent performance. The morphological and colloidal characterization, including field emission scanning electron microscopy (FE-SEM), transmission electron microscopy (TEM), dynamic light scattering (DLS), and zeta potential, is then presented in terms of particle aggregation behavior and its implications for drug release and biological interaction. Subsequently, PL properties are examined to establish the suitability of the Eu^3+^-doped CaMoO_4_ shell for imaging-guided therapy. Finally, drug loading, release kinetics, and in vitro cytotoxicity results are discussed to demonstrate how the physicochemical features of the nanocomposite translate into controlled ZA delivery and enhanced antiproliferative activity against osteosarcoma cells.

### 3.1. FT-IR studies

FT-IR spectrometry was used to identify the functional groups in the structure of the HAp–SiO_2_, HAp–SiO_2_–CaMoO_4_:Eu^3+^, and HAp–SiO_2_–CaMoO_4_:Eu^3+^–ZA. In the FT-IR spectrum of HAp–SiO_2_, the broad band observed in the range of 3050–3700 cm^−1^ is indicative of the stretching vibration of the H-0-H and Si-0-H groups. The peaks observed at 2916–2848 cm^−1^ are attributed to the symmetric and asymmetric stretching vibrations of the aliphatic C-H groups, which originate from unreactive TEOS. The absorption peak observed at 1625 cm^−1^ belongs to in-plane H-O-H bending vibrations. The peak observed at 1457 cm^−1^ indicates the asymmetric stretching vibrations of the carbonyl (CO_3_^2−^) groups in the structure of the HAp [21]. CO_3_^2−^ ions are formed by the adsorption of atmospheric CO_2_ on the surface of the HA nanocomposites [22]. The peaks observed at 1108 cm^−1^ and 1030 cm^−1^ were attributed to stretching vibrations in siloxane (Si-O-Si) bridges. The stretching and bending vibrations of Si-O groups were at 814 cm^−1^ and 468 cm^−1^, respectively [23]. In addition, the asymmetric stretching vibration bands of the phosphate groups (PO_4_^3−^) overlap with the siloxane (Si-O-Si) stretching vibration bands in the range of 964–1290 cm^−1^. Moreover, the asymmetric bending vibration of phosphate (PO_4_^3−^) groups is observed at 563 cm^−1^ [24]. In the FT-IR spectrum of HAp–SiO_2_–CaMoO_4_:Eu^3+^ nanocomposites, the broad band observed at 3460 cm^−1^ is attributed to the asymmetric vibration of the 0-H groups and it overlaps with the asymmetric vibration bands of Si-0-H. The peak at 1633 cm^−1^ belongs to H-O-H bending vibrations. The asymmetric stretching vibration of the carbonyl (CO_3_^2−^) group is observed at 1409 cm^−1^. The sharp peak observed at 1037 cm^−1^ belongs to the stretching vibration band of the silane siloxane (Si-O-Si) bridges. The observed strong doublet peak at 848 cm^−1^ and 801 cm^−1^ is attributed to asymmetric bending vibrations in the MoO_4_ lattices of the tetragon and it overlaps with the Si-O vibrations. The FT-IR spectra of the HAp–CaMoO_4_:Eu^3+^ and HAp–CaMoO_4_:Eu^3+^–ZA nanocomposites are similar to each other because they have the same functional groups. Overall, the FT-IR results confirm the preservation of key functional groups after each synthesis and loading step, indicating the chemical stability of the carrier system, which is essential for reliable drug loading and controlled release performance. The FT-IR spectra of the HAp–SiO_2_, HAp–SiO_2_–CaMoO_4_:Eu^3+^, and HAp–SiO_2_–CaMoO_4_:Eu^3+^–ZA nanocomposites are given in Supplementary Figure S2.

### 3.2. XRD studies

**Figure 1:**
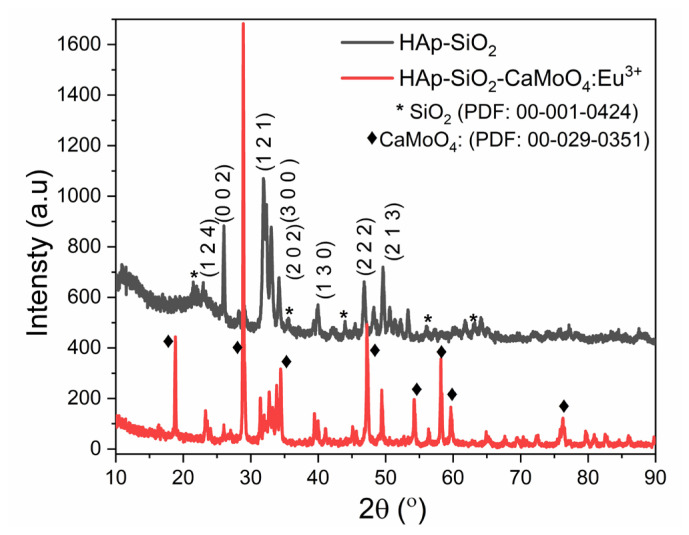
XRD powder pattern of HAp–SiO_2_ and HAp–SiO_2_–CaMoO_4_:Eu^3+^ nanocomposites.

The XRD powder patterns of the HAp–SiO_2_ and HAp–SiO_2_–CaMoO_4_:Eu^3+^ nanocomposites are given in Figure 1. The distinct reflections observed at 2θ values of 22.96°, 26.03°, 31.91°, 33.03°, 34.12°, 39.96°, 46.80°, and 49.58° in the XRD powder pattern of the HAp nanocomposites correspond to (124), (002), (121), (300), (202), (130), (222), and (213) hkl values, respectively. HAp nanocomposites with the Ca_10_(PO_4_)_6_(OH)_2_ phase (PDF card number: 01-080-6199) were crystallized in the hexagonal crystal system and the P63/m 176 space group. The cell parameters, which were calculated using Miller indices for the hexagonal crystal system, were a_(110)_ = 9.5186, c_(002)_ = 7.0218, and c_(004)_ = 7.6809. In the XRD pattern of HAp–SiO_2_, the low bands shown with an asterisk in the range of 20° to 70° indicate the amorphous SiO_2_ structure, which is crystallized in the cubic crystal system and the Fd-3m (227) space group (in the cristobalite β-SiO_2_ phase; PDF card number: 00-001-0424). The reflections observed at 2θ values of 21.46°, 35.59°, 43.91°, 56.07°, and 63.18° in the XRD powder pattern of SiO_2_ correspond to (111), (220), (222), (331), and (422) hkl values, respectively. In the XRD powder pattern of the HAp–SiO_2_–CaMoO_4_:Eu^3+^ nanocomposites, sharp reflections of the CaMoO_4_ layer are also observed (indicated in figures by ♦). CaMoO_4_ crystallizes in the tetragonal crystal system with the I41/a (88) space group (in the powellite phase; PDF card number: 00-029-0351). The reflections observed at 18.84°, 28.88°, 34.47°, 54.25°, 58.18°, 59.64°, and 76.29° 2θ values correspond to hkl values of (101), (112), (200), (116), (312), (224), and (316), respectively. The XRD powder pattern of the HAp–SiO_2_–CaMoO_4_:Eu^3+^ nanocomposites shows that CaMoO_4_ can successfully crystallize on the surface of HAp.

These results confirm the successful formation of a chemically and structurally integrated core–shell system, which is essential for achieving the stable drug loading and reliable luminescent performance required for theranostic applications. This structural integrity provides a solid foundation for the subsequent optical and biological functionalities required for an integrated theranostic platform.

### 3.3. Photoluminescence studies

**Figure 2:**
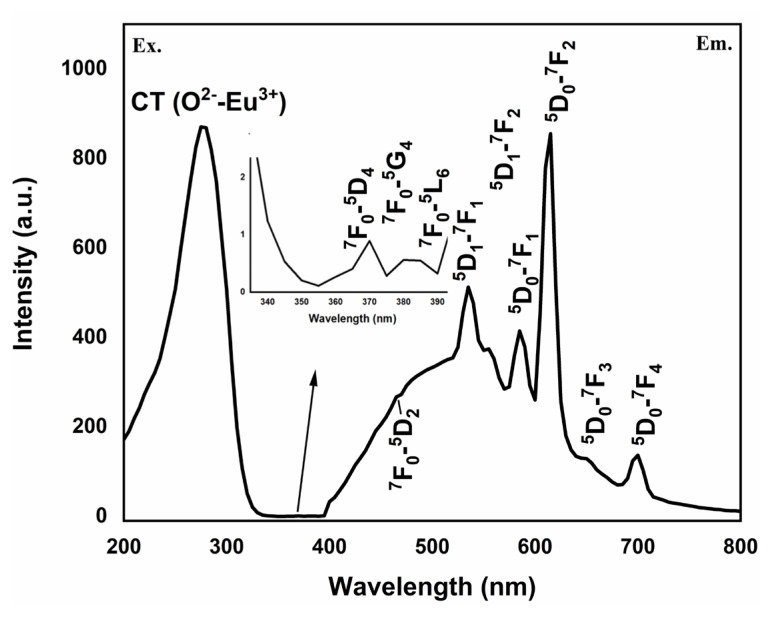
Excitation and photoluminescence spectra of HAp–SiO_2_–CaMoO_4_:Eu^3+^ nanocomposites.

The PL spectra of the HAp–SiO_2_–CaMoO_4_:Eu^3+^ nanocomposites are given in Figure 2. PL measurements were performed at room temperature using a Cary Eclipse fluorescence spectrophotometer (Agilent, Santa Clara, CA, USA) equipped with a solid sample holder. Powdered samples (~0.5 g) were carefully packed into a cylindrical quartz cell to ensure uniform surface contact with the excitation beam. Excitation and emission spectra were collected at a scan rate of 300 nm/min, with slit widths of 5 nm for excitation and 10 nm for emission.

The excitation spectra of the HAp–SiO_2_–CaMoO_4_:Eu^3+^ nanocomposites primarily consist of two parts. The most intense excitation band at 277.5 nm was observed lying in the range of 200–345 nm, while the other part, from 345 to 470 nm, comprises very weak excitation peaks. There are three different mechanisms to explain the charge transfer band (CTB): (i) electron transfer from O_2-_ to Mo^6+^, (ii) intervalence charge transfer due to electron transition from the 4f state of Eu^3+^ to Mo^6+^, or (iii) electron transfer from the 2p orbital of O_2-_ to the vacant 4f orbital of Eu^3+^. The weak peaks observed at wavelengths of 370, 380, 385, and 465 nm belonged to the intraconfigurational 4f–4f transition ^7^F_0_ → ^5^D_4_, ^7^F_0_ → ^5^G_2–6_, ^7^F_0_ → ^5^L_6_, ^7^F_0_ → ^5^D_2_ of the Eu^+3^ ions, respectively [25].

Emission spectra were measured following excitation at 277.5 nm as the most intense CTB, showing ^5^D_0_ → ^7^F_n_ (n = 1, 2, 3, 4) transitions at 585 nm, 615 nm, 650 nm, and 699.5 nm, as well as ^5^D_1_ → ^7^F_n_ (n = 1, 2) transitions at 535 nm and 555 nm. The maximum emission intensity was observed for the ^5^D_0_ → ^7^F_2_ transition, which is the hypersensitive electric dipole transition, reflecting the variation in the bonding environment of Eu^3+^ ions. The ^5^D_0_ → ^7^F_1_ transition is a magnetic dipole transition, while all other transitions are induced electric dipole transitions.

The experimental outcome described here and the assignment of the transitions are consistent with previous studies on Eu^3+^-doped phosphors [26,27], confirming the reproducibility and reliability of the observed PL behavior.

The strong red emission centered at 615 nm demonstrates that the CaMoO_4_:Eu^3+^ shell remains optically active after integration and drug loading, supporting the intended imaging function of the system in therapeutic applications. This confirms that the imaging capability of the system is not compromised by structural integration or drug incorporation, which is a critical requirement for theranostic applications.

### 3.4. Surface morphology and potential analysis

The surface morphologies of the HAp–SiO_2_ and HAp–SiO_2_–CaMoO_4_:Eu^3+^ nanocomposites were examined by FE-SEM and TEM. It was determined that HAp–SiO_2_ nanocomposites were distributed as heterogeneous, hexagonal, long single and clustered nanocomposites in the size range of 182–368 nm (length) × 89.5–113 nm (width) in FE-SEM micrographs. When the TEM micrographs of the HAp–SiO_2_ nanocomposites were examined, it was seen that they were rod-shaped, heterogeneous, and distributed in the size range of 109.29–156.88 nm (length) × 13.40–37.24 nm (width).

**Figure 3:**
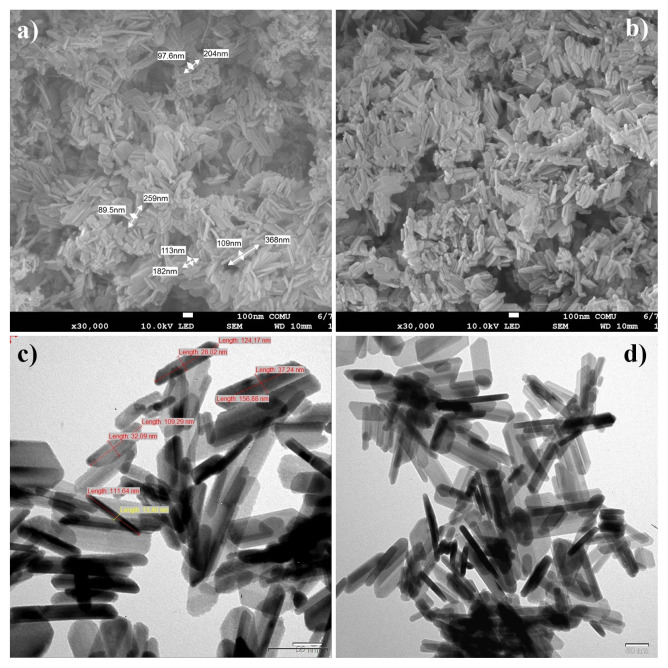
FE-SEM **(a, b)** and TEM micrographs **(c, d)** of HAp–SiO_2_ nanocomposites.

**Figure 4:**
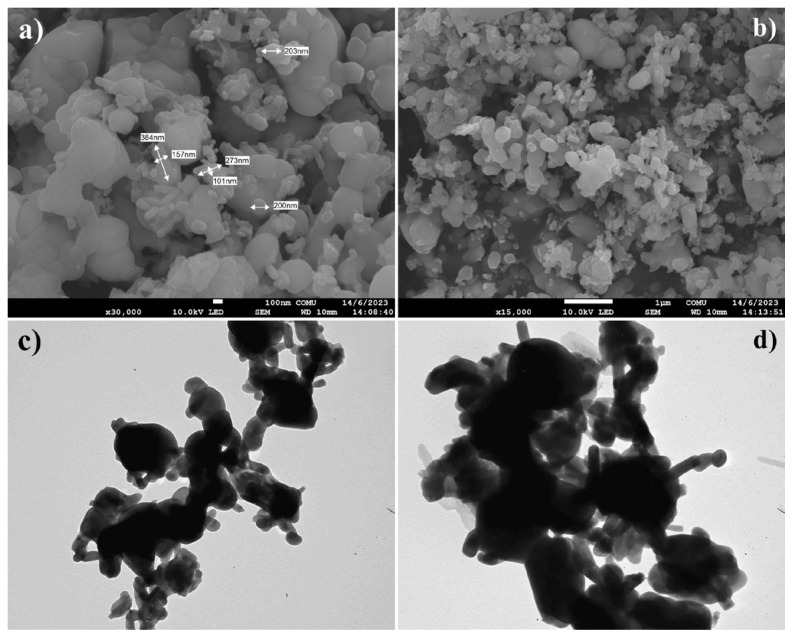
FE-SEM **(a, b)** and TEM **(c, d)** micrographs of HAp–SiO_2_–CaMoO_4_:Eu^3+^ nanocomposites.

In the FE-SEM and TEM micrographs of the HAp–SiO_2_–CaMoO_4_:Eu^3+^ nanocomposites, spherical, heterogeneous, and clustered distributions are observed. In addition, it is seen that HAp–SiO_2_–CaMoO_4_:Eu^3+^ nanocomposites are distributed in the size range of 200–384 nm (length) × 101–157 nm (width) in FE-SEM micrographs. FE-SEM and TEM micrographs of the HAp–SiO_2_ nanocomposites are given in Figure 3, while FE-SEM and TEM micrographs of the HAp–SiO_2_–CaMoO_4_:Eu^3+^ nanocomposites are given in Figure 4.

Particle size distribution measurements of the HAp–SiO_2_ and HAp–SiO_2_–CaMoO_4_:Eu^3+^ nanocomposites were carried out according to the principle of DLS with the aid of a Zeta-sizer (Malvern Instruments, Malvern, UK). All nanocomposites were dispersed in PBS (pH 7.4) to obtain the appropriate scattering intensity. Measurements were taken by averaging three different counting cycles in distilled water at 25 °C and an angle of 90°. The mean particle size of the HAp–SiO_2_ nanocomposites was determined as 1085 nm, and the mean particle size of the HAp–SiO_2_–CaMoO_4_:Eu^3+^ nanocomposites was determined as 1427 nm. The particle size distributions of the HAp–SiO_2_ and HAp–SiO_2_–CaMoO_4_:Eu^3+^ nanocomposites are given in Supplementary Figures S3 and S4, respectively. The zeta potential of the HAp–SiO_2_–CaMoO_4_:Eu^3+^ nanocomposites was measured by averaging three different counting cycles in distilled water at 25 °C and an angle of 90°. The zeta potential of the HAp–SiO_2_–CaMoO_4_:Eu^3+^ nanocomposites was found to be −0.732 mV, −1.77 mV, and −0.401 mV in three different measurements.

This broad morphological variety, ranging from hexagonal or rod-shaped single or clustered HAp–SiO_2_ particles to spherical, clustered HAp–SiO_2_–CaMoO_4_:Eu^3+^ particles, is consistent with literature reports showing that the morphology of HAp and related composites is highly sensitive to factors such as precursor concentration, pH, reaction time, dopant/filler content, nucleation vs. growth kinetics, and agglomeration behavior [28]. In particular, the rod- and hexagonal-shaped morphologies observed in the HAp–SiO_2_ particles align with studies showing that HAp crystals may adopt hexagonal rod or whisker morphology when controlled hydrothermal conditions are applied or when growth is induced on SiO_2_ surfaces [29]. The shift toward spherical and clustered morphology in the HAp–SiO_2_–CaMoO_4_:Eu^3+^ particles can be interpreted in terms of the CaMoO_4_:Eu^3+^ phase acting as a nucleation/growth modifier, as the additional phase likely alters the local supersaturation, surface energy landscape, and aggregation tendency, thereby promoting more isotropic (spherical) growth and enhanced clustering. This is consistent with reports stating that CaMoO_4_ preparations often yield irregular spherical-like nanoparticles and that solvents or cophases significantly affect the morphology [30,31]. Furthermore, the DLS results showing much larger hydrodynamic sizes (~1–1.4 μm) than the nanoscale dimensions obtained by FE-SEM/TEM can be reasonably attributed to particle aggregation (clusters) in suspension, hydration shell effects, and the inherently different measurement principle of DLS (i.e., hydrodynamic diameter vs. physical diameter). The very low absolute zeta potential values (−0.732 to −0.401 mV) for the HAp–SiO_2_–CaMoO_4_:Eu^3+^ particles point to a low surface charge and thus weak electrostatic repulsion, which favors aggregation and clustering in an aqueous medium. Thus, the large DLS sizes are consistent with a clustered/aggregated state in PBS. Similar effects involving disparity between TEM/SEM size and DLS size due to aggregation have been previously discussed in the literature [32].

Although aggregation was observed in aqueous media, such clustering behavior is commonly reported for bioceramic-based nanocomposites and does not preclude their applicability in localized or injectable bone-targeted delivery systems. Moreover, in the context of bone-targeted and locally administered systems, such aggregation may even be advantageous as it can enhance retention at the target site and reduce premature systemic distribution.

### 3.5. Drug release and drug kinetics

**Figure 5:**
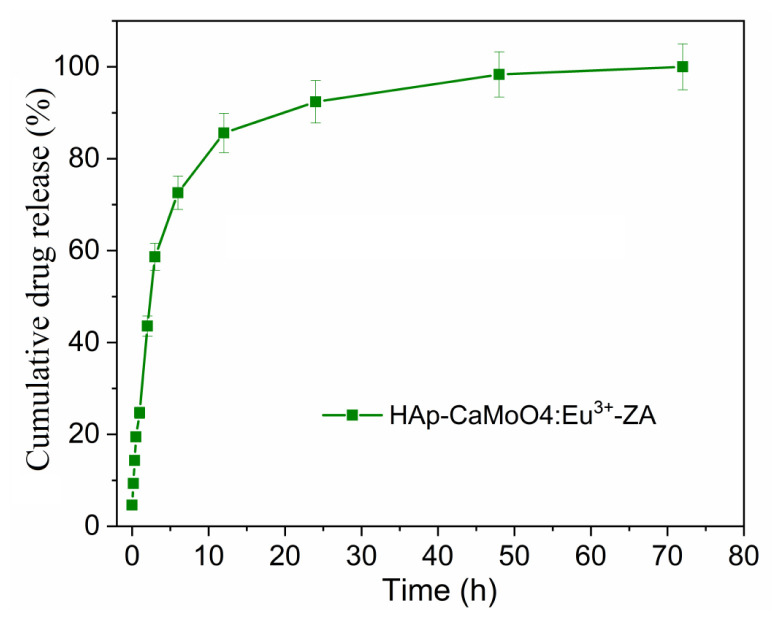
Percentage of cumulative drug release of HAp–SiO_2_–CaMoO4:Eu^3+^–ZA nanocomposites.

The cumulative drug release percentage plot of the HAp–SiO_2_–CaMoO_4_:Eu^3+^–ZA nanocomposites versus time is given in Figure 5.

**Figure 6:**
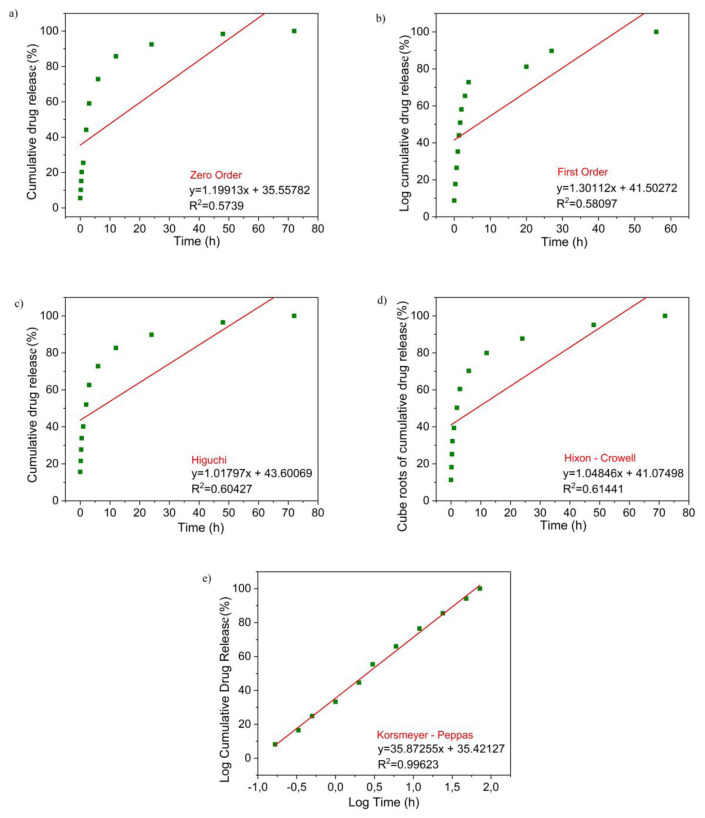
Drug release curves of HAp–SiO_2_–CaMoO4:Eu^3+^–ZA nanocomposites adapted to different kinetic models: **a)** zero order, **b)** first order, **c)** Higuchi model, **d)** Hixon–Crowell model, **e)** Korsmeyer–Peppas model.

From data obtained from the HAp–SiO_2_–CaMoO4:Eu^3+^–ZA nanocomposites at pH 7.4 and 37 °C in PBS, drug release was calculated according to various kinetic models including zero-order, first-order, Higuchi, Hixson–Crowell, and Korsmeyer–Peppas models. Drug release curves of the HAp–SiO_2_–CaMoO4:Eu^3+^–ZA nanocomposites adapted to different kinetic models are given in Figure 6. The correlation value (R_2_) was determined with the help of the slope of the curves in the graphs obtained for each kinetic model. The model with the highest R^2^ value for the HAp–SiO_2_–CaMoO4:Eu^3+^–ZA nanocomposites was found to be the Korsmeyer–Peppas model. The correlation value (R^2^) for Korsmeyer–Peppas modeling of the HAp–SiO_2_–CaMoO4:Eu^3+^–ZA nanocomposites was 0.99623; the n value was found to be 0.83. The n value in the Korsmeyer–Peppas model is the diffusion exponent and it characterizes the release mechanism of the drug.

These results show that the drug release of the HAp–SiO_2_–CaMoO4:Eu^3+^–ZA nanocomposites entails non-Fickian transport (0.45 < n = 0.89) according to the Korsmeyer–Peppas model [33,34]. In summary, the HAp–SiO_2_–CaMoO4:Eu^3+^–ZA nanocomposites comply with the Korsmeyer–Peppas model in terms of drug release kinetics.

The sustained non-Fickian release behavior directly supports the design objective of prolonged local delivery of ZA, thereby reducing systemic exposure and potential side effects. This release profile is particularly suitable for osteosarcoma treatment, where sustained local exposure to ZA is desired to maximize antitumor efficacy while minimizing off-target effects.

### 3.6. Cell viability studies

**Figure 7:**
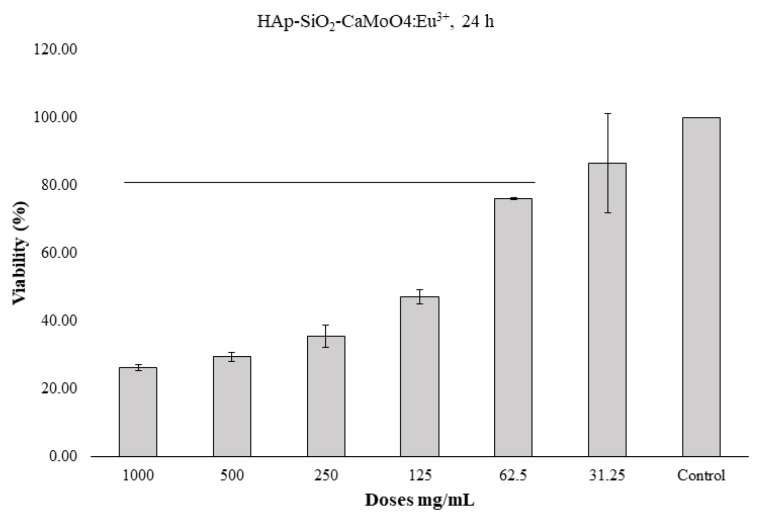
HAp–SiO_2_–CaMoO_4_:Eu^3+^, IC_50-Saos-2_ = 81.73, 24-h MTT, *: p < 0.05 (figure prepared using GraphPad Prism 6.0).

**Figure 9:**
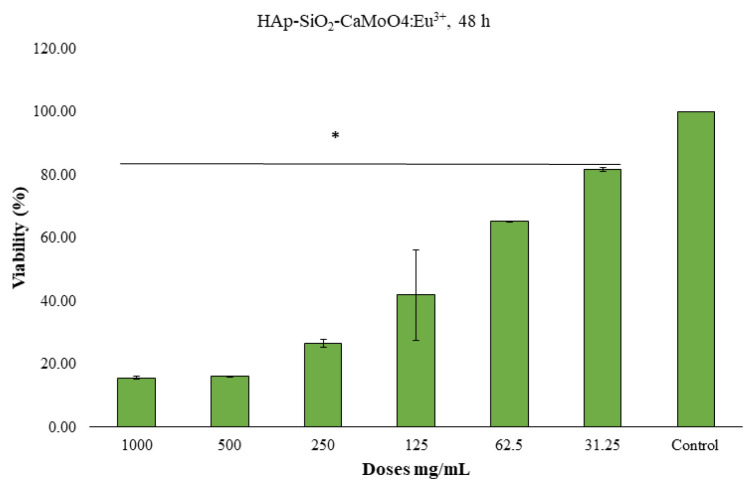
HAp–SiO_2_–CaMoO_4_:Eu^3+^, IC_50-Saos-2_ = 69.82, 48-h MTT, *: p < 0.05 (figure prepared using GraphPad Prism 6.0).

**Figure 10:**
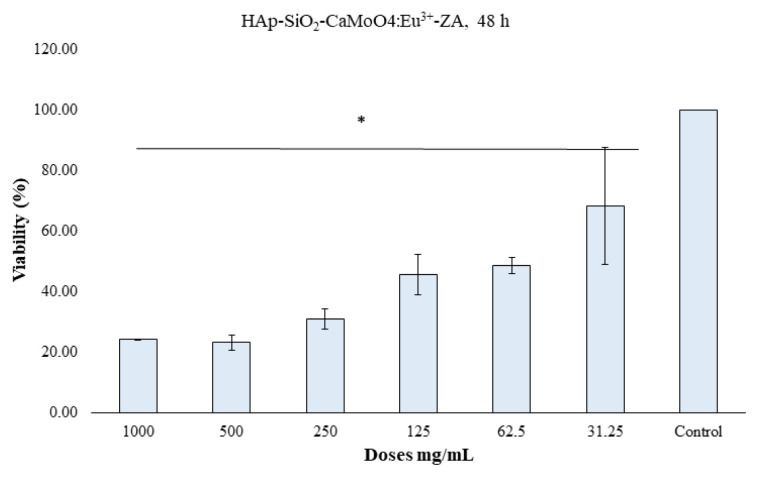
HAp–SiO_2_–CaMoO_4_:Eu^3+^–ZA, IC_50-Saos-2_ = 56.33, 48-h MTT, *: p < 0.05 (figure prepared using GraphPad Prism 6.0).

The MTT method was used to determine the 24-h and 48-h cytotoxic effects of the HAp–SiO_2_–CaMoO_4_:Eu^3+^ and HAp–SiO_2_–CaMoO_4_:Eu^3+^–ZA nanocomposites against Saos-2 cells cultured from osteosarcoma tissues. The results obtained are given as dose-dependent percentage viabilities in Figures 7–10. In the graphs summarizing the cytotoxicity results (Figures 7–10), the nanocomposites were found to be cytotoxic and antiproliferative for the Saos-2 cells at all concentrations in columns marked with stars. As a result of the application of the HAp–SiO_2_–CaMoO_4_:Eu^3+^ and HAp–SiO_2_–CaMoO_4_:Eu^3+^–ZA nanocomposites to Saos-2 cells for 24 h, the IC_50_ value was calculated as 81.73 μM and 80.07 μM, respectively. After applying HAp–SiO_2_–CaMoO_4_:Eu^3+^ and HAp-SiO_2_–CaMoO_4_:Eu^3+^–ZA nanocomposites to Saos-2 cells for 48 h, the IC_50_ values were 69.82 μM and 56.33 μM, respectively. Thus, the HAp–SiO_2_–CaMoO_4_:Eu^3+^ and HAp–SiO_2_–CaMoO_4_:Eu^3+^–ZA nanocomposites had dose-dependent cytotoxic effects. According to these results, the cytotoxic effect of the HAp–SiO_2_–CaMoO_4_:Eu^3+^ nanocomposites is increased in drug-loaded HAp–SiO_2_–CaMoO_4_:Eu^3+^–ZA nanocomposites. In particular, the 48-h cytotoxic effect of the HAp–SiO_2_–CaMoO_4_:Eu^3+^–ZA nanocomposites (IC_50_ of 56.33 μM for Saos-2 cells) had a significant impact on the viability and differentiation of the Saos-2 cells, which are osteosarcoma cells, and these particles were more effective against the Saos-2 cells due to the cellular release of ZA with high toxicity. The tested nanocomposites were found to be effectively cytotoxic for Saos-2 cells. It was previously reported in the literature that HAp nanocomposites have negligible cytotoxicity for Saos-2 cells [35,36]. In addition, HAp–APTES–BT and HAp–APTES–BT–ZA nanocomposites obtained by functionalizing the surface of HAp nanocomposites showed cytotoxic effects even at low doses [36].

The enhanced cytotoxicity observed for the ZA-loaded nanocomposites compared to unloaded carriers demonstrates that the therapeutic function of the system was preserved and effectively coupled to the carrier design. Together with the controlled release behavior and confirmed optical activity, these cytotoxicity results demonstrate that the designed nanocomposite successfully integrates diagnostic and therapeutic functions within a single carrier system.

## 4 Conclusion

In this study, Eu^3+^-doped CaMoO_4_ functionalized HAp–SiO_2_ (HAp–SiO_2_–CaMoO_4_:Eu^3+^) nanocomposites were successfully synthesized and comprehensively characterized, demonstrating their potential as multifunctional theranostic platforms. In line with the primary objective of developing an integrated system capable of simultaneous drug delivery and imaging for osteosarcoma therapy, each structural, optical, and biological result was evaluated in terms of functional contributions.

FT-IR and XRD analyses confirmed the coexistence of biocompatible HAp, stabilizing SiO_2_, and luminescent CaMoO_4_:Eu^3+^ phases, indicating successful formation of well-integrated core–shell structures. This structural integrity is critical for ensuring both stable drug incorporation within the HAp–SiO_2_ core and reliable optical performance from the CaMoO_4_:Eu^3+^ shell, thereby directly supporting the intended theranostic function. FE-SEM and TEM studies revealed diverse morphologies, as the HAp–SiO_2_ exhibited heterogeneous hexagonal and rod-shaped particles, while the addition of CaMoO_4_:Eu^3+^ led to spherical and clustered structures, consistent with literature reports on morphology modulation by dopants and cophases. Such morphological evolution is particularly relevant to drug-carrier performance, as it influences surface area, aggregation behavior, and ultimately drug loading and release characteristics. DLS and zeta potential measurements further demonstrated particle aggregation in aqueous media, highlighting the importance of colloidal stability for biomedical applications. These findings explain the observed release kinetics and emphasize the need to balance structural complexity with dispersion behavior when designing nanocarriers for physiological environments. The PL studies confirmed a strong red emission at 615 nm due to hypersensitive Eu^3+^ transitions, directly fulfilling the imaging component of this study’s aim and validating the suitability of the system for luminescence-guided therapy. ZA was efficiently loaded into the nanocomposites via supercritical CO_2_ (25 °C, 200 bar) with entrapment efficiency of 99.97%, and in vitro release studies showed an initial burst (43.56% in 2 h) followed by sustained release, consistent with the Korsmeyer–Peppas model (n = 0.83), indicating a non-Fickian diffusion mechanism. This release behavior confirms that the designed core–shell architecture effectively regulates ZA delivery, aligning with the study’s objective of minimizing systemic side effects while maintaining therapeutic efficacy. Cytotoxicity assays performed against Saos-2 osteosarcoma cells demonstrated dose- and time-dependent antiproliferative effects. Notably, ZA-loaded nanocomposites exhibited significantly enhanced cytotoxicity (IC_50_ = 56.33 μM at 48 h), while unloaded HAp–SiO_2_–CaMoO_4_:Eu^3+^ showed minimal toxicity, clearly linking the observed biological response to controlled ZA delivery rather than intrinsic material toxicity. Taken together, these results demonstrate that HAp–SiO_2_–CaMoO_4_:Eu^3+^–ZA nanocomposites integrate drug delivery, controlled release, bioimaging, and biocompatibility within a single platform, offering a versatile approach for targeted osteosarcoma therapy. By explicitly correlating the physicochemical structure, optical behavior, drug-release kinetics, and biological response with the overarching therapeutic and diagnostic purpose, this study has provided a coherent framework for the rational design of next-generation theranostic nanomaterials. The ability to modulate particle morphology, tune the PL, and achieve high drug loading underscores the potential of this multifunctional nanocomposite as a next-generation theranostic material, bridging experimental outcomes with clinically oriented design considerations and real-time imaging-assisted therapy.

## Supplemantry materials

Figure S1 Calibration curve of ZA at different concentrations (1 mg/mL, 3 mg/mL, 5 mg/mL, 7 mg/mL and 9 mg/mL).

Figure S2 FTIR Spectra of HAp-SiO_2_, HAp-SiO_2_-CaMoO_4_:Eu^3+^ and HAp-SiO_2_-CaMoO_4_:Eu^3+^-ZA nanocomposites.

Figure S3 The particle size distribution of the HAp-SiO_2_ nanocomposites.

Figure S4 The particle size distribution of the HAp-SiO_2_-CaMoO_4_:Eu^3+^ nanocomposites.

## Figures and Tables

**Figure 8:**
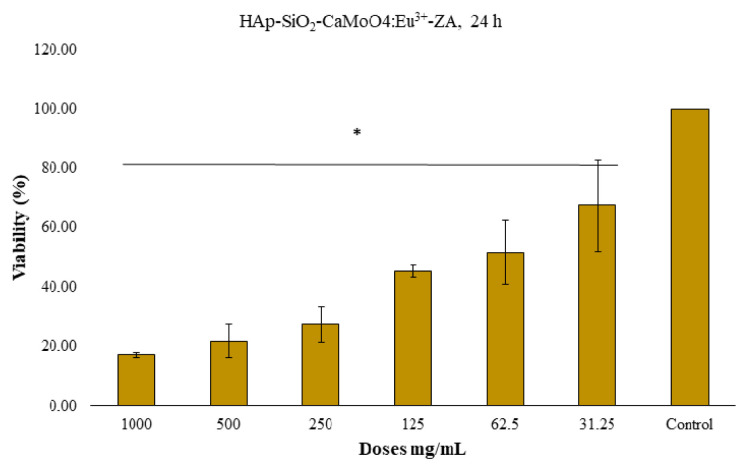
HAp–SiO_2_–CaMoO_4_:Eu^3+^–ZA, IC_50-Saos-2_ = 80.07, 24-h MTT, *: p < 0.05 (figure prepared using GraphPad Prism 6.0).

**Figure 8:**
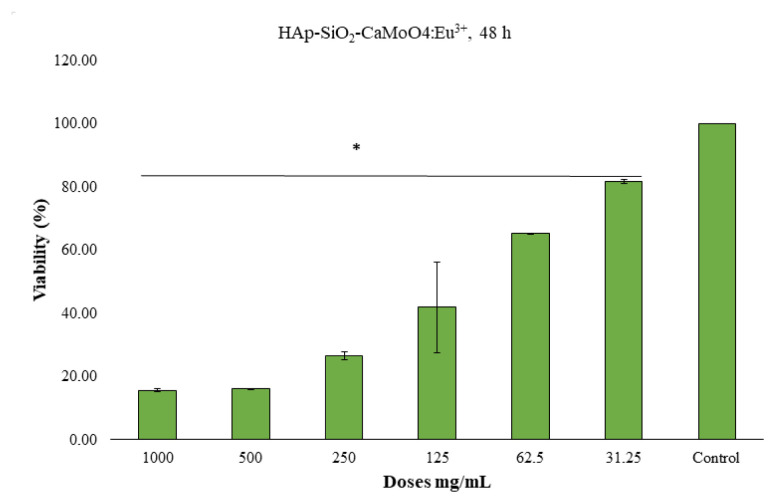
HAp–SiO_2_–CaMoO_4_:Eu^3+^–ZA, IC_50-Saos-2_ = 80.07, 24-h MTT, *: p < 0.05 (figure prepared using GraphPad Prism 6.0).
