# Supplementary figures and images for: Zoledronic acid-loaded HAp–SiO2–CaMoO4:Eu3+ with luminescent properties as a novel drug delivery system
Source: Turk J Chem. 2025 Dec 26;50(1):61–74. doi: 10.55730/1300-0527.3780 (PMC12965784; doi:10.55730/1300-0527.3780)

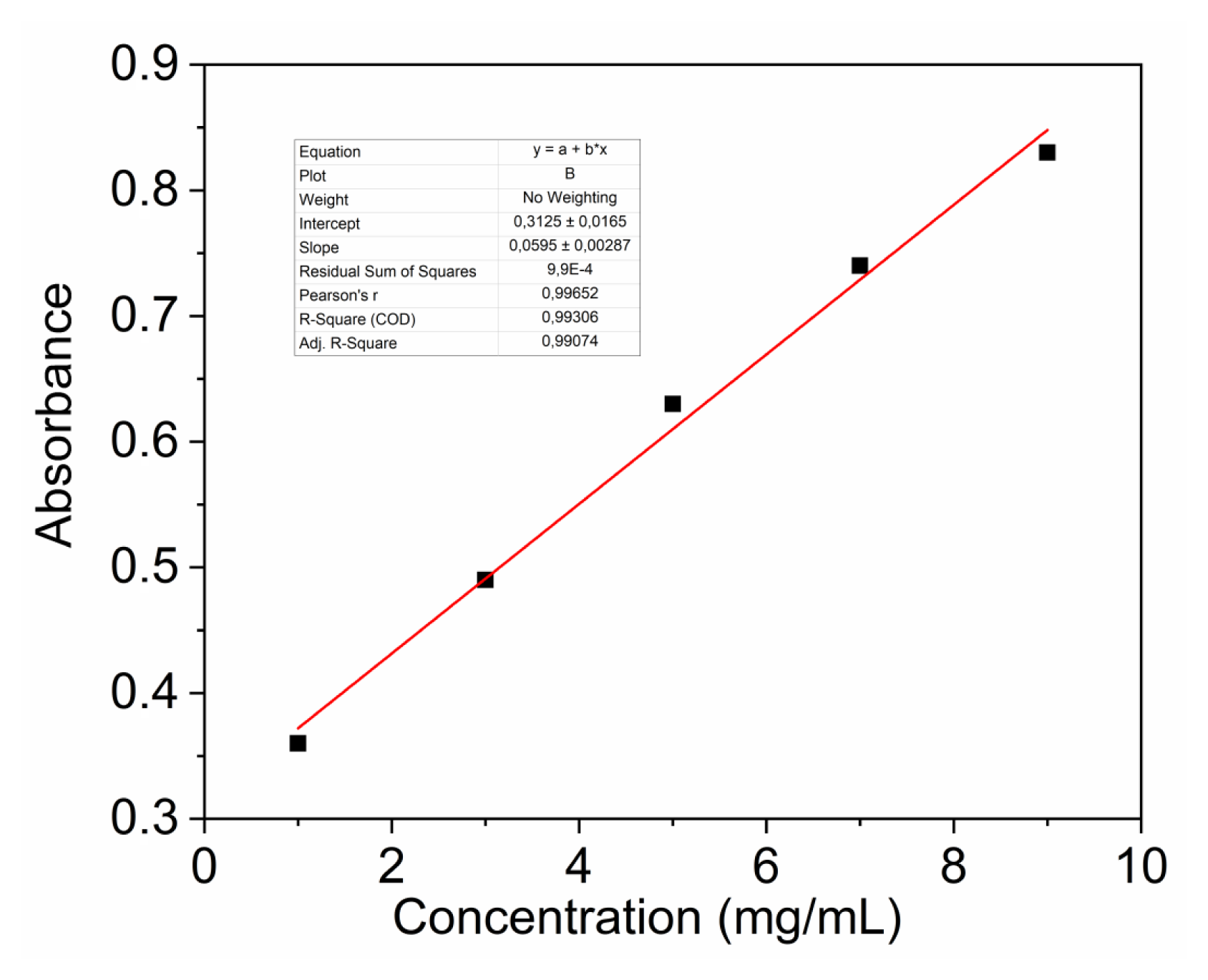

Supplement: Figure S1 — Calibration curve of ZA at different concentrations (1 mg/mL, 3 mg/mL, 5 mg/mL, 7 mg/mL and 9 mg/mL). [file tjc-50-01-61s1.tif]

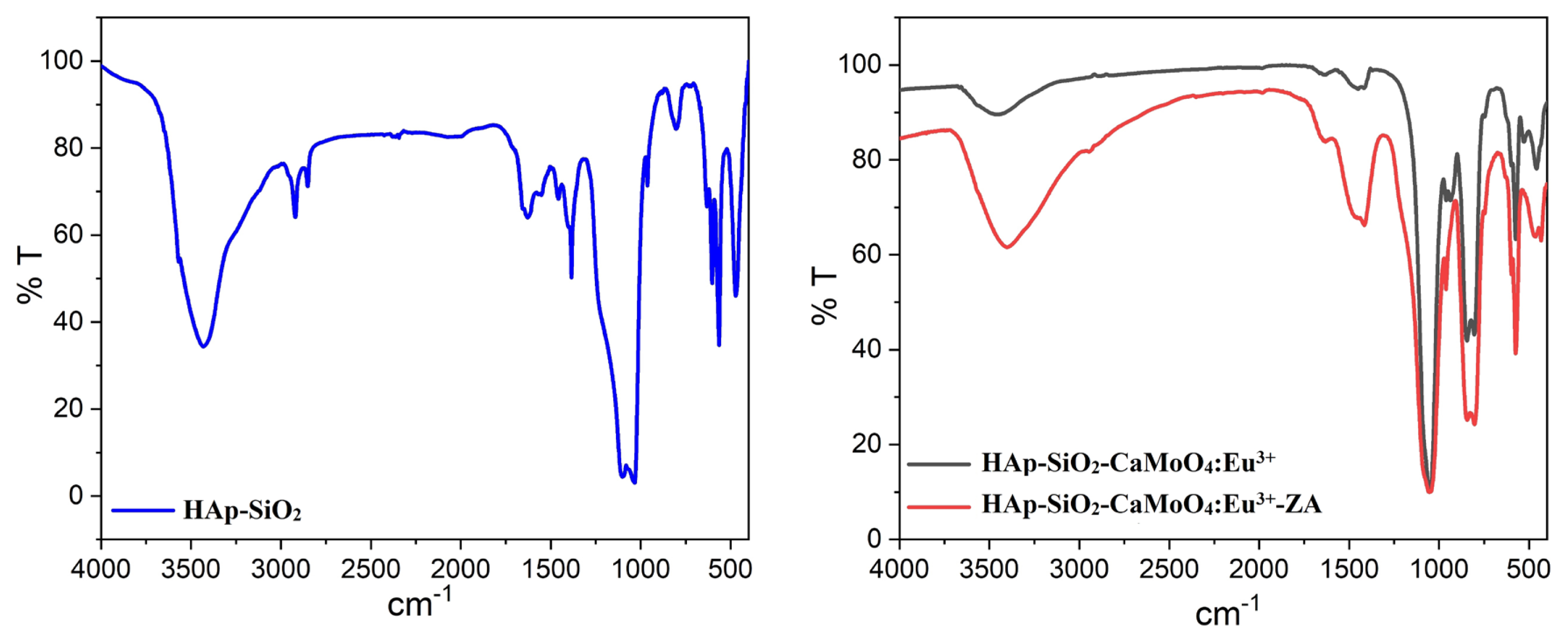

Supplement: Figure S2 — FTIR Spectra of HAp-SiO2, HAp-SiO2-CaMoO4:Eu3+ and HAp-SiO2-CaMoO4:Eu3+-ZA nanocomposites. [file tjc-50-01-61s2.tif]

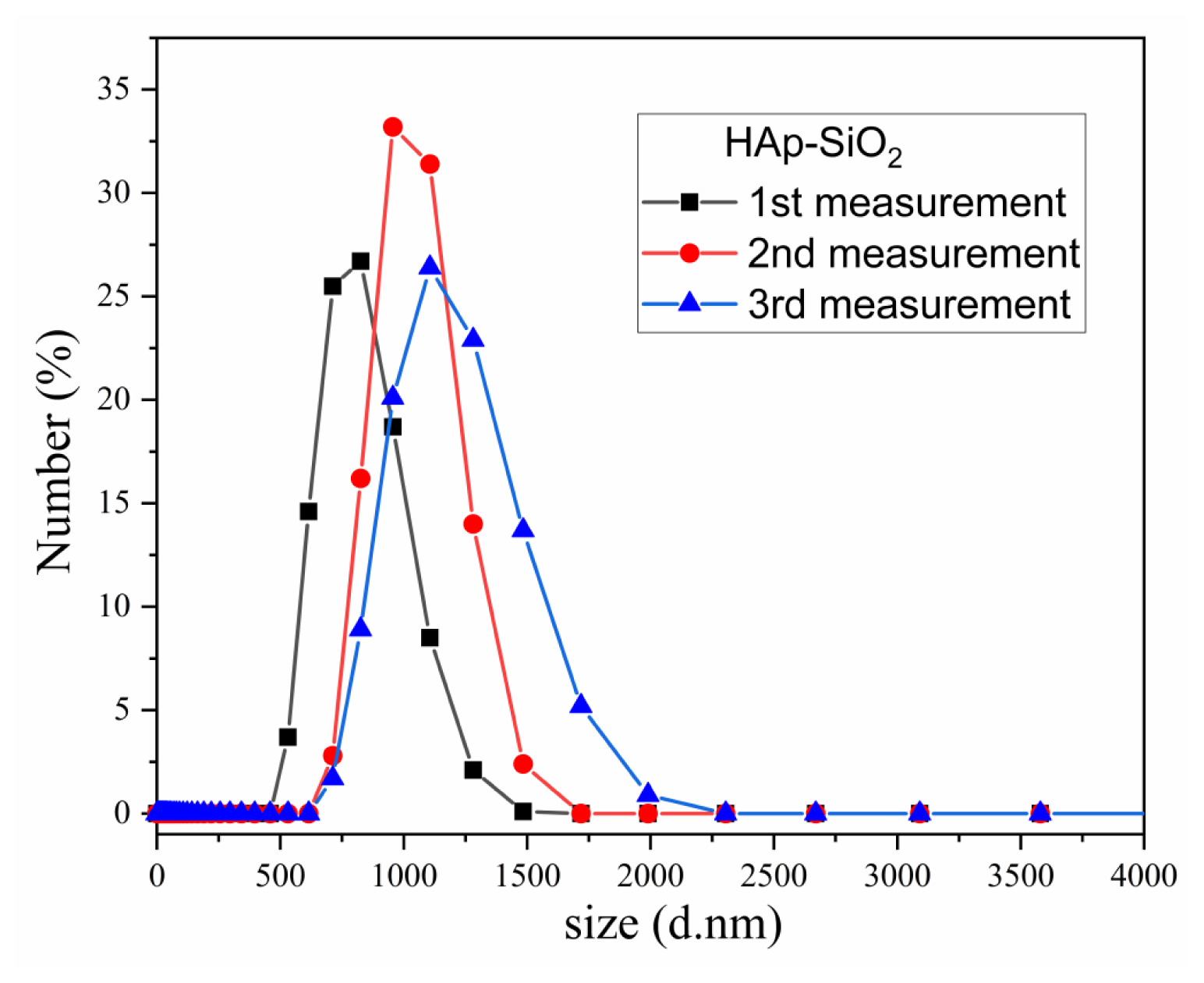

Supplement: Figure S3 — The particle size distribution of the HAp-SiO2 nanocomposites. [file tjc-50-01-61s3.tif]

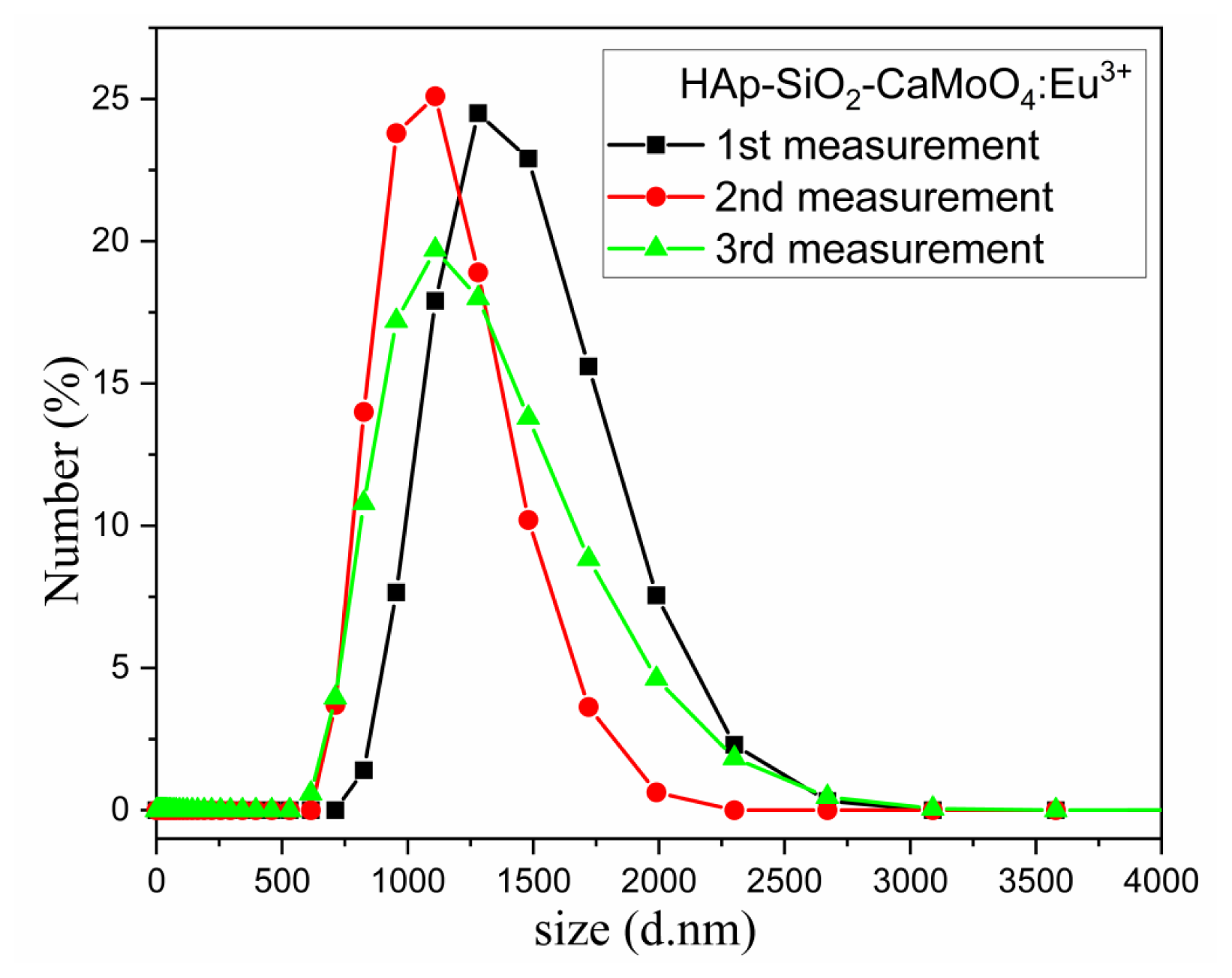

Supplement: Figure S4 — The particle size distribution of the HAp-SiO2-CaMoO4:Eu3+ nanocomposites. [file tjc-50-01-61s4.tif]
